# Treatment of open-angle glaucoma and ocular hypertension with preservative-free tafluprost/timolol fixed-dose combination therapy: 6 case reports and clinical outcomes

**DOI:** 10.1186/s12886-022-02361-7

**Published:** 2022-04-02

**Authors:** E. Ansari, S. Chappiti, J. Pavicic-Astalos, J. C. Pinto-Bonilla, I. Riva, M. Sacchi, F. Saénz-Francés

**Affiliations:** 1 Maidstone and Tunbridge Wells NHS Trust, Tunbridge Wells, UK; 2 Institute of Medical Sciences, Christ Church Canterbury University, Kent, UK; 3 Royal Wolverhampton Hospitals NHS Trust Eye Infirmary, New Cross Hospital, Wolverhampton, UK; 4 Institute of Eye Surgery, Waterford, Ireland; 5 Complejo Hospitalario de Navarra, Pamplona, Spain; 6 Istituto Clinico Sant’Anna, Brescia, Italy; 7 Clinica Oculistica Universitaria, Università degli Studi di Milano, Ospedale San Giuseppe Milano, Milan, Italy; 8 Hospital Clínico Universitario San Carlos, Universidad Complutense, Madrid, Spain

**Keywords:** Open-angle glaucoma, Ocular hypertension, Fixed-dose combination, Preservative-free topical medication, Tafluprost/timolol, Ocular surface disease, Benzalkonium chloride, BAK, Case report

## Abstract

**Background:**

Treatment of open angle glaucoma (OAG) and/or ocular hypertension (OHT) focuses on achievement of target intraocular pressure (IOP), with the objective of slowing disease progression. However, ocular surface health is an important consideration in the optimization of treatment. We report 6 patient cases in which enhanced IOP control was achieved following appropriate management of ocular surface inflammation and a therapeutic switch to the preservative-free (PF) tafluprost (0.0015%)/timolol (0.5%) fixed-dose combination (FC).

**Case presentation:**

Six patient cases, aged 48–74 years, presented with OAG or OHT. Each patient had signs and symptoms of ocular surface disease (OSD). Cases 1–3 were each receiving maximal medical therapy for OAG; regimens comprising prostaglandin analogue (PGA), β-blocker, carbonic anhydrase inhibitor (CAI) and α-2 agonist agents (including treatments containing preservative agent). Cases 1 and 2 reported IOP values ≥23 mmHg in each eye, and wide IOP fluctuations were identified when reviewing patient data concerning case 3 (11–20 mmHg). Maximal therapy was ceased and PF tafluprost/timolol FC was initiated, after which the signs and symptoms of OSD were improved and IOP was reduced (≤18 mmHg for cases 1–3) and stabilized. Cases 4 and 5 were diagnosed with OAG and case 6 had OHT. Each had symptoms and signs of OSD and were treated with a preserved PGA monotherapy (latanoprost 0.005% or bimatoprost 0.03%). At presentation, IOP was 24 mmHg in both eyes (case 4), ≥18 mmHg (case 5) and ≥ 22 mmHg (case 6). Following a switch to the PF tafluprost/timolol FC, OSD symptoms were improved and IOP was 14 mmHg (both eyes; case 4), ≤14 mmHg (case 5) and 16 mmHg (both eyes; case 6).

**Conclusions:**

In addition to IOP-lowering efficacy, approaches to the management of OAG and OHT should consider the impact of treatment tolerability and the susceptibility of these patients to OSD. The presence of ocular surface inflammation appears to be detrimental to adherence and therefore to the effectiveness of topical medications. Addressing OSD through the use of PF FC formations, such as the PF tafluprost/timolol FC, reduces exposure to potentially toxic agents and facilitates improvements in IOP control.

## Background

Elevated intraocular pressure (IOP) is the only proven modifiable risk factor for the development and progression of open-angle glaucoma (OAG) leading to permanent sight loss [1]. As a chronic condition, glaucoma typically requires long-term use of topical IOP-lowering medications and patients may be elderly, with a complex range of comorbidities [2]. Prevalence of ocular surface disease (OSD) is higher among people with glaucoma, compared with the general population, making tolerability an important consideration for ophthalmologists when selecting appropriate IOP-lowering therapies [2–5]. Glaucoma treatments associated with ocular surface toxicity, such as those containing the preservative agent benzalkonium chloride (BAK), exacerbate OSD, reduce quality of life (QoL) and often result in poor compliance and/or adherence with subsequent worsening of glaucoma signs/symptoms [6–8]. Ideally, topical glaucoma treatments, should provide a balance of powerful efficacy alongside an acceptable tolerability profile [9]. Recent data indicate that treatments that are better tolerated at the ocular surface and result in less OSD may be associated with further reductions in IOP among people with glaucoma, possibly as a result of improved treatment adherence and reduced inflammation at the site of instillation, which allows IOP-lowering medications to act more effectively [5, 7, 9–14]. The European Glaucoma Society (EGS) guidelines emphasize that treatment should consider patient QoL from the outset [2]. However, in clinical practice, OAG treatment escalation/intensification will often focus primarily on the goal of lowering IOP or gaining better IOP control (reducing fluctuations) to slow disease progression and visual deterioration, with tolerability considered as a secondary issue. More holistic approaches that also consider ocular surface health (particularly when evidence of OSD or treatment-related toxicity is present) may provide better long-term outcomes as obstacles to efficacy, such as poor treatment adherence and tolerability issues, are lessened [5, 7, 13, 14]. The authors estimate (based upon experience within their own centers and the literature in this area) that approximately 30–60% of OAG patients seen at ophthalmology clinics may demonstrate signs or symptoms of treatment-related OSD that should act as a flag for therapeutic review or a switch to a preservative-free (PF) formulation [15, 16].

Here, we report 6 clinical cases of primary open angle glaucoma (POAG) or ocular hypertension (OHT) from ophthalmology clinics across Europe, in which OSD, treatment tolerability, IOP control and compliance were improved following a switch to the PF prostaglandin analogue (PGA) and β-blocker fixed-dose combination (FC) of tafluprost (0.0015%)/timolol (0.5%). These cases highlight the important clinical indicators and symptoms relating to ocular surface health that should be considered or monitored during the therapeutic pathway so that local treatment-related toxicities can be promptly identified and prioritized during disease management, in addition to IOP lowering and signs of disease progression. The cases also emphasize the importance of active listening and awareness of non-verbal cues in fostering a successful doctor-patient relationship.

## Case presentation

### Treatment simplification from maximal therapy with a preservative-free fixed-dose combination therapy

#### Case 1

A 68-year-old man with a diagnosis of POAG was referred for filtration surgery. On examination, IOP was 26 mmHg in both the right and left eye. Right eye mean deviation (MD) was − 5.15 dB and pattern standard deviation (PSD) was 5.73 dB. Left eye (MD) was − 7.28 dB and PSD was 6.78 dB. Treatment duration at this stage was 8 years. Treatment had been gradually increased to maximal medical therapy, comprising an α-2 agonist, carbonic anhydrase inhibitor (CAI), β-blocker and PGA, with the aim of achieving target IOP. He had been prescribed brimonidine 0.2% (twice daily), brinzolamide 0.1%/timolol 0.5% (FC twice daily) and travoprost 0.004% (once daily). All treatments prescribed at this stage contained BAK.

The patient presented with conjunctival hyperemia, reduced tear break-up time (TBUT) (3 s) and superficial punctate keratitis (SPK). Confocal microscopy showed infiltration of inflammatory cells at the ocular surface. Optical coherence tomography (OCT) analysis revealed structural progression of disease over 6 years, mainly in the left eye. Between 2013 and 2019, retinal nerve fiber layer (RNFL) thickness reduced by approximately 1.25 μm/year (from 75 μm to 70 μm) in the right eye and by 1.97 μm/year (from 79 μm to 67 μm) in the left eye. Superior RNFL thickness was reduced by 3.89 μm/year (from 107 μm to 86 μm), which was accompanied by functional deterioration and visual field loss.

Following a washout period of 3 weeks, during which hydrocortisone drops were applied (3 times daily) and oral acetazolamide (250 mg twice daily) was given, diurnal IOP remained consistent with that of maximal therapy. Based upon published data from randomized controlled trials (RCT) and real-world observational studies, a decision was made to simplify treatment through a switch to PF tafluprost/timolol FC (once daily) [17–22].

**Fig. 1:**
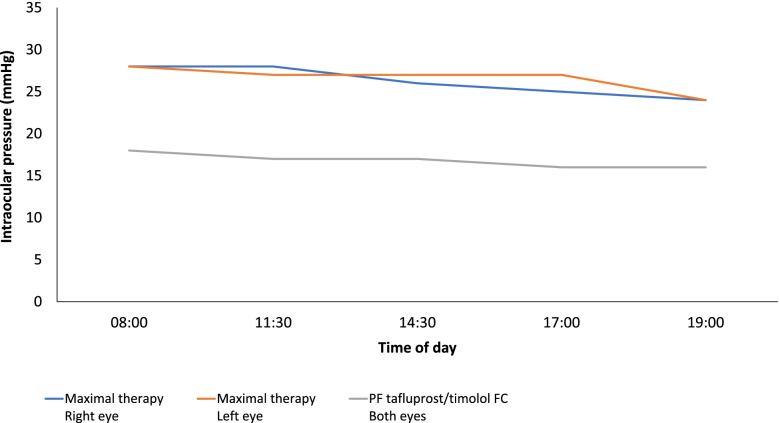
Case 1: Diurnal IOP curves with maximal therapy* and following switch to PF tafluprost/timolol FC. *Maximal therapy: brimonidine 0.2% (twice daily), brinzolamide 1%/timolol 0.5% fixed combination (twice daily) and travoprost 0.004% (once daily)

**Fig. 2:**
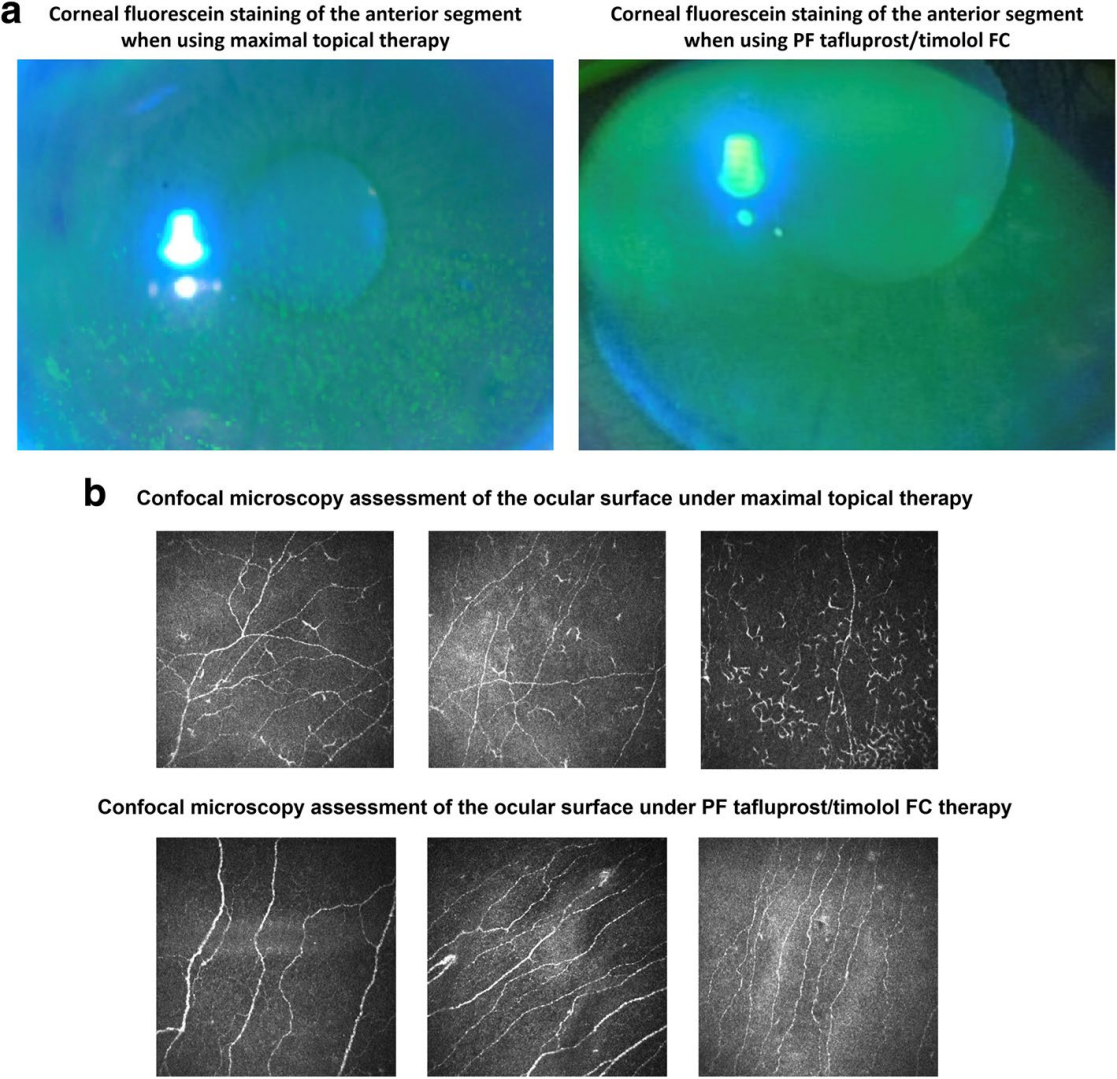
**a**. Case 1: corneal fluorescein staining of the anterior segment under maximal topical therapy and after initiating PF tafluprost/timolol FC treatment; **b**. Case 1: confocal microscopy images from the anterior segment under maximal topical therapy and after initiating PF tafluprost/timolol FC treatment

During follow-up (4 weeks), the mean IOP was reduced to 17 mmHg (Fig. 1) and improvements were observed regarding the severity of conjunctival hyperemia, keratopathy and SPK. Corneal fluorescein staining (CFS) in the anterior segment was reduced following initiation of PF tafluprost/timolol FC therapy (Fig. 2a) and confocal microscopy revealed a marked reduction in the number of inflammatory cells at the surface of the eye (Fig. 2b) and POAG disease was stabilized, with the rate of progression reducing over the following 2 years.

#### Case 2

A 67 year-old Caucasian woman with POAG presented following adverse events (AEs) associated with her prior medication, which had resulted in the development of OSD. IOP was 23 mmHg (right eye) and 24 mmHg (left eye) and she showed bilateral field defects. Signs of OSD comprised meibomian gland dysfunction (MGD), reduced TBUT (< 5 s), aqueous tear deficiency and SPK. Right eye MD was − 7.0 dB and left eye MD was − 23.0 dB. The patient had been on topical therapy for 26 months. She was currently receiving maximal topical therapy containing BAK-preserved agents: bimatoprost 0.03% (once daily), dorzolamide/timolol FC (twice daily) and brimonidine 0.2% (twice daily).

All topical glaucoma treatments were ceased and a 2-week washout period commenced, during which a lid care regimen was given alongside acetazolamide 250 mg (twice daily), PF dexamethasone drops (4 times daily) and tear substitutes (administered as required). OSD continued to improve and stabilize. Selective laser trabeculoplasty (SLT) was performed, after which PF tafluprost/timolol FC treatment was commenced. IOP was reduced to 14 mmHg (right eye) and 18 mmHg (left eye) at 8-week follow-up.

#### Case 3

A 67-year-old man with POAG and previous cataract extraction in the left eye presented for review. He was using preserved latanoprost 0.005% eye drops (once daily) in both eyes from the time of diagnosis, 5 years before. Ophthalmological examination revealed a best-corrected visual acuity of 20/25 in the right eye and 20/20 in the left eye, and a central corneal thickness of 536 μm and 539 μm, in the right and left eye, respectively. Slight OSD was detected with CFS (Oxford Grade Scale: 1–2). Visual field test was within the normal limits in the right eye (VFI: 100%; MD: + 0.63 dB; PSD: 1.38 dB) but showed an initial inferior arcuate scotoma in the left eye (VFI: 94%; MD: − 3.45 dB; PSD: 5.48 dB), matching a superior peripapillary nerve fiber defect at the OCT analysis (Heidelberg Spectralis OCT, Heidelberg Engineering Ltd., Heidelberg, Germany). RNFL supero-nasal thickness was 69 μm and RNFL supero-temporal thickness was 80 μm. Daytime IOP curve with Goldman applanation tonometry found an IOP peak at 10 am of 16 mmHg (right eye) and 19 mmHg (left eye).

To address IOP control, a preserved FC of β-blocker and CAI was added to the existing latanoprost therapy. After 3 months, a daytime IOP curve was repeated, demonstrating an IOP peak of 13 mmHg and 13.5 mmHg, in the right and in the left eye, respectively, at 10 am. IOP control was considered satisfactory, despite a worsening of the OSD (CFS Oxford Grade scale: 2–3), and the patient was advised to carry on the new treatment.

**Fig. 3:**
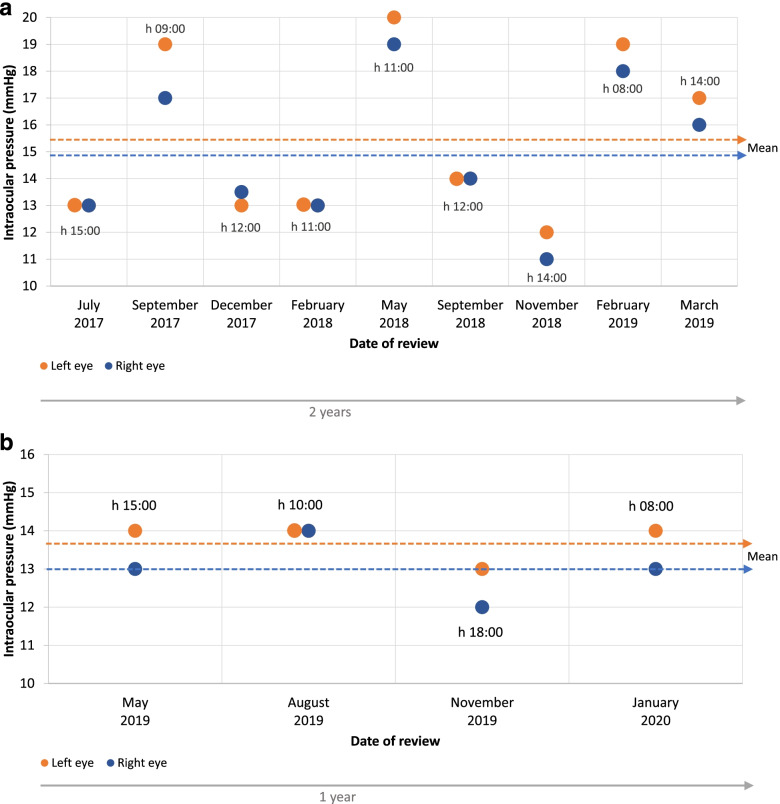
**a**. Case 3: IOP data over 2-year follow-up on β-blocker/CAI FC and latanoprost therapy; **b**. Case 3: IOP data over 1-year follow-up on PF tafluprost/timolol FC therapy

The patient was regularly followed-up in the next months. After 2 years, a slight worsening of the visual field in the left eye was confirmed (VFI: 89%; MD: − 4.04 dB; PSD: 5.43 dB), despite IOP being apparently controlled. Retrospective chart review revealed a mean calculated IOP (over the previous 2 years) of 14.95 mmHg and 15.5 mmHg, in the right and in the left eye, respectively. However, considerable fluctuation of IOP values was detected in both eyes (IOP range: 11–20 mmHg) (Fig. 3a). When specifically asked, the patient admitted to poor adherence/compliance with his prescribed treatment regimen, due to local symptoms (i.e. red eye, itching and burning sensation), suggesting an underestimation of OSD symptoms at previous visits, in favor of an excessively low target IOP.

Treatment was switched to the PF tafluprost/timolol FC in both eyes. After 4 weeks, a new daytime IOP curve revealed a peak IOP of 14 mmHg (right eye) and 15 mmHg (left eye). Target IOP was deemed to be reached. Improvements were observed in both the OSD (CFS Oxford Grade Scale: 0–1) and the treatment compliance. Follow-up over a 1-year period showed that visual field and IOP remained stable, with a marked reduction of IOP fluctuation (Fig. 3b, IOP range: 12–14 mmHg). Mean IOP over the 1-year follow-up was 13 mmHg (right eye) and 13.75 mmHg (left eye).

### Stepping up to a preservative-free fixed-dose combination therapy from preserved prostaglandin analogue monotherapy

#### Case 4

A 74-year-old Caucasian man, diagnosed with POAG, was referred by his optician to a specialist ophthalmology clinic due to inadequate IOP control. Although not reported within the referral documentation, his main ocular symptoms comprised red sore eyes, watering and blurred vision. He presented with mixed blepharitis, reduced TBUT (< 5 s), injected conjunctivae and bilateral SPK. CFS (Oxford Grade Scale) was grade 3. Visual acuity (VA) was 6/9 unaided (right and left eye). IOP was 24 mmHg in both eyes and early bilateral field defects were present.

At referral, the patient was receiving preserved latanoprost 0.005% eye drops (once daily). Right eye MD was − 5.7 dB and left eye MD was − 4.8 dB. The patient had been on topical therapy for less than 6 months.

Treatment was stepped up to PF tafluprost/timolol FC (once daily) and advice was given on eyelid care, with doxycycline 50 mg (daily for 4 weeks) and eyelid wipes prescribed to address blepharitis. At 8-week follow-up, IOP was reduced to 14 mmHg (both eyes), ocular surface inflammation was reduced, CFS (Oxford Grade Scale) was grade 1, TBUT was increased to 8 s and vision was reported to be improved. The patient commented that his adherence had improved as the medication felt more comfortable upon administration, compared with the previous treatment.

#### Case 5

**Fig. 4:**
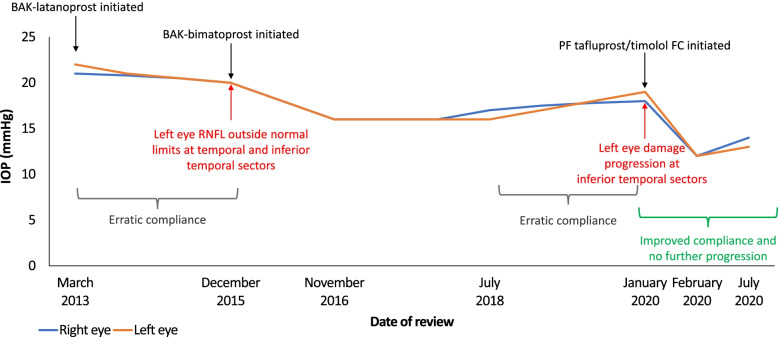
Case 5: IOP change over 7-year follow up

A 63-year-old male Caucasian with moderate myopia in both eyes was referred for assessment due to a family history of OAG. Left visual field was limited and RNFL assessments showed borderline defects at the inferior temporal sector in the left eye. IOP was 21 mmHg (right eye) and 22 mmHg (left eye). Corneal thickness was 511 μm (right eye) and 508 μm (left eye). In the right eye, VFI was 99%, MD was − 0.63 dB, PSD was + 1.27 dB and PGH parameter was outside of normal levels. In the left eye, VFI was 98%, MD was − 0.93 dB, PSD was + 1.91 dB and PGH parameter was outside of normal as well. Left visual field was limited and the patient had a family history of glaucoma, with reduced corneal thickness as an added risk factor. Figure 4 shows the patient journey regarding change in IOP over a 7-year follow up. BAK-preserved latanoprost 0.005% monotherapy (once daily), over a period of 2 years, provided a modest reduction in IOP to 20 mmHg (both eyes) and RNFL assessments revealed slight progression at the temporal and inferior-temporal sectors (left eye). The patient reported erratic compliance due to discomfort on application of therapy.

Treatment was switched to preserved bimatoprost 0.01% monotherapy and assessments at 1-year revealed that compliance was adequate, IOP was 16 mmHg (both eyes) and the RNFL defect at the inferior temporal sector was stabilized. The patient reported eye discomfort that was graded as 5 using a 10-point visual analogue scale (VAS), where 0 was no discomfort and 10 was worst possible discomfort. TBUT was 7 s (both eyes) and ocular surface disease index (OSDI) score was 18. The OSDI score defines the severity of OSD; normal (0–12 points), mild (13–22 points), moderate (23–32 points) or severe (33–100 points). Artificial tears were prescribed to address the dry eye symptoms. After a further 2 years, IOP control was maintained (17 mmHg in the right eye and 16 mmHg in the left eye), but symptoms of OSD had worsened. TBUT was 5 s (both eyes) and OSDI score was 19, while discomfort was graded as 3 (VAS 0–10). Treatment remained unchanged due to the apparent stabilization of IOP and progressive disease.

After a further 2 years, IOP had begun to rise, measuring 18 mmHg (right eye) and 19 mmHg (left eye), and progressive RFNL damage was detected at the inferior temporal sector (Fig. 4). In the right eye, VFI was 91%, MD was − 0.63 dB, PSD was + 1.27 dB and PGH parameter was out of normal range. In the left eye, VFI 89%, MD − 4.32 dB, PSD + 9.22 dB and PGH parameter out of normal range. TBUT was 4 s, OSDI score was 22 and discomfort score had risen to 8. Again, the patient admitted to poor compliance due to discomfort upon instillation of preserved bimatoprost.

Treatment was changed to PF tafluprost/timolol FC and artificial tear use was continued (as required). After just 4 weeks, dry eye symptoms had begun to improve. TBUT was 7 s, OSDI score was 12, and discomfort score had dropped to 4. IOP had also reduced to 12 mmHg (both eyes). Follow-up at 6 months revealed that the patient’s condition had stabilized. TBUT was 7 s (both eyes), OSDI score remained at 12 and discomfort score was 6. IOP was 14 mmHg (right eye) and 13 mmHg (left eye) and no further RNFL damage was observed.

#### Case 6

A female 48-year-old Caucasian with OHT was referred via her optometrist following a high IOP measurement; 33 mmHg (right eye) and 32 mmHg (left eye). She had mild (untreated) arterial hypertension, VA was 6/12 corrected (right and left eye), she was a contact lens wearer and complained of irritable eyes. On examination, IOP was 26 mmHg (right eye) and 30 mmHg (left eye) and preserved latanoprost 0.005% monotherapy was prescribed. IOP was subsequently reduced to 22 mmHg (right eye) and 26 mmHg (left eye) at 1-year follow-up. MD was + 1.25 dB in the right eye and + 0.75 dB in the left eye. The cup-to-disc (CD) ratio was 0.4 in both eyes. However, she appeared particularly anxious about her condition at her subsequent annual review with signs of MGD and conjunctival hyperemia. She also reported having sore eyes and felt unable to wear contact lenses and make-up or to take part in physical activity.

Treatment was switched to PF tafluprost/timolol FC. Within 4 weeks, the patient’s conjunctival hyperemia had resolved and symptoms of OSD were improved. In addition, IOP at 4-week follow-up was 16 mmHg (right and left eyes). Follow-up over 2 years demonstrated that IOP was stabilized and symptoms of OSD remained mild, with occasional artificial tear use. She reported that she had felt able to resume regular swimming sessions and had returned to her previous active lifestyle. Following her positive treatment outcomes with PF tafluprost/timolol FC, the patient offered to take part in an educational video to share her experience.

## Discussion and conclusions

The cases presented emphasize the importance of considering tolerability and ocular surface toxicities in the treatment of OHT and OAG. The cases highlight three common themes associated with glaucoma management, which deserve exploration as they reflect reoccurring clinical scenarios faced by ophthalmologists across the globe and may impact both treatment outcomes and patient QoL. Firstly, as shown in cases 1–3, it is possible to achieve a healthier ocular surface, improved treatment compliance or adherence and reliable IOP control with fewer topical medications; using a ‘less is more’ approach. These principles are aligned with recommendations from the EGS, American Glaucoma Society (AGS) and Asia Pacific Glaucoma Society (APGS) guidelines, which recommend that therapy should use the least amount of medications to achieve the target response, while considering inconvenience to the patient, QoL, cost and side effects [2, 23, 24]. Second, OSD is common in people with glaucoma and, as highlighted in EGS guidelines, preferred practice must include concurrent evaluation of the ocular surface alongside parameters for glaucoma/OHT during the initial clinical assessment [2, 5, 7, 9–14]. Thirdly, approaches to management that focus on the achievement of target IOP without consideration for the patient’s ocular comfort or OSD may likely result in poor adherence and suboptimal clinical outcomes [5, 7, 14].

These cases showed that, whether stepping down from maximal therapy or intensifying treatment from first-line PGA monotherapy, a switch to the PF tafluprost/timolol FC may be associated with improvements in OSD symptoms and enhanced or maintained IOP control. These outcomes reflect data from large RCTs and real-world observational studies in which a change to PF tafluprost/timolol FC resulted in significant IOP reductions and low rates of toxicity-related adverse events (e.g. conjunctival hyperemia, dry eye), regardless of whether patients were previously treated with a monotherapy or combination therapies [17–22].

A simplified PF and FC therapy that included fewer topical therapies, compared with maximal therapy, resulted in reduced and/or stabilized IOP and slowing of progressive disease (cases 1, 2 and 3). As demonstrated in case 3, phasing of IOP data over time can provide a useful indication of true IOP control, identifying fluctuations that may not be immediately obvious from a single measurement taken during office hours. Both short-term and long-term fluctuations in IOP may be predictive of poor treatment compliance or glaucoma progression and are worthy of consideration in the holistic management of the disease [25, 26].

Markers of OSD and patient-reported tolerability were improved when maximal therapy was replaced by the PF tafluprost/timolol FC. Case 1 showed that fewer inflammatory cells were evident with confocal microscopy following a change to PF tafluprost/timolol FC treatment, probably due to reduced ocular surface exposure to preservative-containing agents that are commonly associated with irritation and inflammation [3, 4, 27–32]. Studies have also suggested that the active agents themselves may play a role in triggering OSD [17]. Bourne et al. (2019) showed that a switch to PF tafluprost/timolol FC from either preserved or PF bimatoprost/timolol FC resulted in improvements regarding the signs and symptoms of OSD while IOP control was maintained [17]. The wash out period used in cases 1 and 2 may have been helpful in reducing preservative-induced inflammation. Although real-world studies suggest that improvements in IOP control and subjective ocular symptoms may be possible when switching directly to the PF tafluprost/timolol FC, a wash out period that includes a short course of steroids may accelerate the resolution of OSD symptoms (particularly when Brimonidine or other adrenergic agents have been used) and prepare the ocular surface for the introduction of a new glaucoma treatment [32].

Taking account of clinical characteristics, corneal thickness, myopia or family history can reveal risk factors for glaucoma progression. However, filtering surgery is less likely to have satisfactory outcomes for those at highest risk unless OSD is managed and controlled [5, 30]. As demonstrated in case 2, approaches that optimize ocular surface health alongside IOP-lowering efficacy may enable topical therapies to be used in combination with non-invasive techniques, such as SLT, to slow disease progression and delay or even prevent the requirement for more invasive techniques. Topical glaucoma treatments containing the PGA tafluprost have been shown to improve ocular hemodynamics and increase mean ocular perfusion pressure, and these properties may have contributed to the outcomes seen in case 2 [33, 34].

Cases 4, 5 and 6 showed that patients experiencing poor IOP control and tolerability issues due to BAK-containing PGA monotherapy (bimatoprost and latanoprost) benefited from treatment escalation to the PF tafluprost/timolol FC. In each case, IOP was reduced or stabilized and markers of OSD were improved. This experience reflects the findings of the recently published VISIONARY study, which showed that patients stepping up to PF tafluprost/timolol FC from β-blocker or PGA monotherapy demonstrated significant reductions in IOP from Week 4 that were maintained over 6 months alongside reductions in CFS grade and conjunctival hyperemia [18].

Patient cases 3, 4 and 5 each acknowledged that they had struggled with compliance or adherence due to discomfort associated with administration of glaucoma therapies. Despite the development of devices that aim to monitor adherence with glaucoma treatment regimens, evidence suggests that such technologies are seldom used in practice and the most common way to assess compliance is through questioning and discussion with the patient [35]. This approach relies on the development of a trusting and honest doctor-patient relationship, in which the patient feels that their individual needs and QoL are considered and prioritized. Noticing non-verbal cues and behavioral indicators of anxiety or discomfort can be important in identifying whether the person may have concerns about their current therapy or might be struggling with adherence [36, 37]. Interventions that focus on individualized care and holistic assessment of healthcare needs have been shown to improve adherence in glaucoma therapy and may reduce IOP fluctuations in people with OHT [38].

Existing QoL measures used in glaucoma therapy focus on physical symptoms and functioning only, providing little assistance for clinicians seeking to understand the emotional and social consequences of the disease as well as the impact on a person’s motivation and ability to adhere to treatment [39]. People with glaucoma or OHT will not be aware of changes in their IOP but will be conscious of OSD symptoms and the limitations they place on their daily lives. Asking the patient about the impact that their symptoms may be having on their QoL can reveal issues that might have otherwise gone unnoticed or be worsened by inappropriate treatment.

Although limited in number, the patient cases presented in this paper aim to illustrate common clinical situations faced by ophthalmologists in routine management of glaucoma and OHT. The cases presented focus only on outcomes with topical therapies and further examples examining the impact of tolerability and OSD management on filtration surgery success may be of value in future papers. However, the cases discussed here represent the heterogenous nature of these patients and the challenges faced by clinicians in real-world clinical practice, where a patient-centered approach should balance both IOP control and tolerability to optimize outcomes for each individual.

In the cases discussed, a switch to the PF tafluprost/timolol FC from either maximal therapy or first-line treatments was accompanied by improvements in IOP-lowering efficacy and markers of OSD. The cases emphasize the importance of considering ocular surface inflammation when seeking to optimize glaucoma and OHT outcomes. Where inflammation or OSD are evident, a switch to a treatment with a more acceptable tolerability profile that reduces ocular surface exposure to potentially toxic agents, (e.g. BAK) may provide better long-term IOP control alongside improvements in patient QoL.

## Data Availability

All data generated or analyzed during this study are included in this published article. Further information is available from the authors upon reasonable request.

## Abbreviations

AGS: American Glaucoma Society
APGS: Asia Pacific Glaucoma Society
BAK: Benzalkonium chloride
BPH: Benign prostatic hyperplasia
CAI: Carbonic anhydrase inhibitor
EGS: European Glaucoma Society
FC: Fixed combination
IOP: Intraocular pressure
MD: Mean deviation
MGD: Meibomian gland dysfunction
MI: Myocardial infarction
OAG: Open angle glaucoma
OCT: Optical coherence tomography
OHT: Ocular hypertension
OSD: Ocular surface disease
OSDI: Ocular surface disease index
PF: Preservative free
PGA: Prostaglandin analogue
POAG: Primary open angle glaucoma
PSD: Pattern standard deviation
QoL: Quality of life
RNFL: Retinal nerve fiber layer
TBUT: Tear film break-up time
SLT: Selective laser trabeculoplasty
SPK: Superficial punctate keratitis
VA: Visual acuity
VFI: Visual filed index
